# Supplementary material for: Effect of cirrhosis on prognosis in patients with acute-on-chronic liver failure: a systematic review and meta-analysis
Source: PeerJ. 2025 Sep 25;13:e20049. doi: 10.7717/peerj.20049 (PMC12476863; doi:10.7717/peerj.20049)
Supplement: Supplemental Information 2 [file peerj-13-20049-s002.docx]

1. **The rationale for conducting the systematic review / meta-analysis.**

Acute-on-chronic liver failure has a notably high mortality rate of approximately 50% within 90 days, and currently lacks effective treatment options (1). More importantly, there exists a significant controversy in the literature regarding the role of cirrhosis as a prognostic factor in ACLF. While some research indicates that cirrhosis can independently predict three-month mortality in ACLF patients, other studies suggest that cirrhosis has no significant influence on short-term mortality (2-4). This conflicting evidence creates uncertainty in clinical practice. Given that early diagnosis and accurate prognosis represent the greatest potential for improving survival in ACLF patients, there is a clear need to systematically evaluate and synthesize the available evidence to determine whether cirrhosis truly serves as an independent prognostic factor.

1. **The contribution that it makes to knowledge in light of previously published related reports, including other meta-analyses and systematic reviews.**

This meta-analysis makes several contributions to the existing knowledge base. First and foremost, it addresses a specific research question that has not been previously examined through systematic review methodology. While there is one published systematic review and meta-analysis that investigated the incidence and major risk factors of ACLF (5), no prior meta-analyses have specifically focused on evaluating whether cirrhosis serves as an independent prognostic factor for ACLF mortality. This makes the current study the first comprehensive synthesis of evidence on this particular question.

The analysis is particularly timely and valuable given the contradictory findings in current literature. Some studies have demonstrated that cirrhosis can independently predict three-month mortality in ACLF patients, while others have found no significant impact on short-term mortality. By systematically integrating these conflicting findings, this meta-analysis will provide more robust and reliable evidence regarding the prognostic value of cirrhosis in ACLF patients. This knowledge is especially crucial considering that more than 25% of cirrhosis patients are present with ACLF symptoms upon admission (6).

The findings from this study will have important practical implications for clinical practice and healthcare policy. If cirrhosis is confirmed as an independent prognostic factor, this information could be incorporated into risk stratification strategies and prognostic models for ACLF patients. Conversely, if cirrhosis is found not to be independently associated with mortality, this would suggest that other factors should be prioritized in prognostic assessment. Either outcome would provide valuable evidence-based guidance for clinicians in their decision-making process and for policymakers in developing public health strategies for ACLF management. This contribution is particularly meaningful given the high mortality rate of ACLF and the current lack of effective treatments (1), where accurate prognostication becomes crucial for optimal patient care.

Reference list:

1. Arroyo V, Moreau R, Jalan R. Acute-on-Chronic Liver Failure. N Engl J Med. 2020;382(22):2137-45.

2. Li N, Huang C, Yu KK, Lu Q, Shi GF, Zheng JM. Validation of prognostic scores to predict short-term mortality in patients with HBV-related acute-on-chronic liver failure: The CLIF-C OF is superior to MELD, CLIF SOFA, and CLIF-C ACLF. Medicine (Baltimore). 2017;96(17):e6802.

3. Wang J, Ma K, Han M, Guo W, Huang J, Yang D, et al. Nucleoside analogs prevent disease progression in HBV-related acute-on-chronic liver failure: validation of the TPPM model. Hepatol Int. 2014;8(1):64-71.

4. Yang WB, Chen EQ, Bi HX, Bai L, Chen XB, Feng P, et al. Different models in predicting the short-term prognosis of patients with hepatitis B virus-related acute-on-chronic liver failure. Ann Hepatol. 2012;11(3):311-9.

5. Aggarwal A, Biswas S, Arora U, Vaishnav M, Shenoy A, Swaroop S, et al. Definitions, Etiologies, and Outcomes of Acute on Chronic Liver Failure: A Systematic Review and Meta-analysis. Clin Gastroenterol Hepatol. 2024;22(11):2199-210.e25.

6. Hernaez R, Solà E, Moreau R, Ginès P. Acute-on-chronic liver failure: an update. Gut. 2017;66(3):541-53.
