# Supplementary material for: Effect of cirrhosis on prognosis in patients with acute-on-chronic liver failure: a systematic review and meta-analysis
Source: PeerJ. 2025 Sep 25;13:e20049. doi: 10.7717/peerj.20049 (PMC12476863; doi:10.7717/peerj.20049)
Supplement: Supplemental Information 3 [file peerj-13-20049-s003.docx]

**Table S1.** **The search strategy (Pubmed)**

| Search number | Query | Results |
| --- | --- | --- |
| 1 | "Liver Cirrhosis"[Mesh] | 107,449 |
| 2 | (((Liver Cirrhosis[Title/Abstract]) OR (Cirrhosis[Title/Abstract])) OR (Liver Fibrosis[Title/Abstract])) OR (Hepatic Fibrosis[Title/Abstract]) | 140,548 |
| 3 | ("Liver Cirrhosis"[Mesh]) OR ((((Liver Cirrhosis[Title/Abstract]) OR (Cirrhosis[Title/Abstract])) OR (Liver Fibrosis[Title/Abstract])) OR (Hepatic Fibrosis[Title/Abstract])) | 169,868 |
| 4 | "Liver Failure"[Mesh] | 31,197 |
| 5 | (((Liver Failure[Title/Abstract]) OR (Hepatic Failure[Title/Abstract])) OR (Acute-On-Chronic Liver Failure[Title/Abstract])) OR (ACLF[Title/Abstract]) | 34,743 |
| 6 | ("Liver Failure"[Mesh]) OR ((((Liver Failure[Title/Abstract]) OR (Hepatic Failure[Title/Abstract])) OR (Acute-On-Chronic Liver Failure[Title/Abstract])) OR (ACLF[Title/Abstract])) | 52,602 |
| 7 | (("Liver Cirrhosis"[Mesh]) OR ((((Liver Cirrhosis[Title/Abstract]) OR (Cirrhosis[Title/Abstract])) OR (Liver Fibrosis[Title/Abstract])) OR (Hepatic Fibrosis[Title/Abstract]))) AND (("Liver Failure"[Mesh]) OR ((((Liver Failure[Title/Abstract]) OR (Hepatic Failure[Title/Abstract])) OR (Acute-On-Chronic Liver Failure[Title/Abstract])) OR (ACLF[Title/Abstract]))) | 13,506 |
| 8 | ((((death rate) OR (mortality)) OR (survival rate)) OR (survival)) OR (Prognos) | 2,926,671 |
| 9 | ((("Liver Cirrhosis"[Mesh]) OR ((((Liver Cirrhosis[Title/Abstract]) OR (Cirrhosis[Title/Abstract])) OR (Liver Fibrosis[Title/Abstract])) OR (Hepatic Fibrosis[Title/Abstract]))) AND (("Liver Failure"[Mesh]) OR ((((Liver Failure[Title/Abstract]) OR (Hepatic Failure[Title/Abstract])) OR (Acute-On-Chronic Liver Failure[Title/Abstract])) OR (ACLF[Title/Abstract])))) AND (((((death rate) OR (mortality)) OR (survival rate)) OR (survival)) OR (Prognos)) | 5,301 |

**The search strategy (Cochrane library)**

| Search number | Query | Results |
| --- | --- | --- |
| 1 | (‘Liver Cirrhosis’ OR ‘Cirrhosis’ OR ‘liver fibrosis’ OR ‘hepatic fibrosis’):ab,ti,kw | 14,913 |
| 2 | (‘Liver Failure’ OR ‘hepatic failure’ OR ‘acute on chronic liver failure’ OR ‘ACLF’):ab,ti,kw | 9,460 |
| 3 | (‘death rate’ OR ‘mortality’ OR ‘survival rate’ OR ‘survival’):ab,ti,kw | 240,229 |
| 4 | #1 AND #2 AND #3 | 907 |

**The search strategy (Embase)**

| Search number | Query | Results |
| --- | --- | --- |
| 1 | 'liver cirrhosis':ab,ti,kw OR 'cirrhosis':ab,ti,kw OR 'liver fibrosis':ab,ti,kw OR 'hepatic fibrosis':ab,ti,kw | 227,819 |
| 2 | 'liver failure':ab,ti,kw OR 'hepatic failure':ab,ti,kw OR 'acute on chronic liver failure':ab,ti,kw OR 'aclf':ab,ti,kw | 57,672 |
| 3 | 'death rate':ab,ti,kw OR 'mortality':ab,ti,kw OR 'survival rate':ab,ti,kw OR 'survival':ab,ti,kw | 3,266,206 |
| 4 | #1 AND #2 AND #3 | 5,788 |
